# Worse capecitabine treatment outcome in patients with a low skeletal muscle mass is not explained by altered pharmacokinetics

**DOI:** 10.1002/cam4.4038

**Published:** 2021-06-14

**Authors:** Laura Molenaar‐Kuijsten, Bart Albertus Wilhelmus Jacobs, Sophie Alberdine Kurk, Anne Maria May, Thomas Petrus Catharina Dorlo, Jacob Hendrik Beijnen, Neeltje Steeghs, Alwin Dagmar Redmar Huitema

**Affiliations:** ^1^ Department of Pharmacy & Pharmacology The Netherlands Cancer Institute ‐ Antoni van Leeuwenhoek Amsterdam The Netherlands; ^2^ Department of Radiation Oncology University Medical Center Utrecht Utrecht University Utrecht The Netherlands; ^3^ Julius Center for Health Sciences and Primary Care University Medical Center Utrecht Utrecht University Utrecht The Netherlands; ^4^ Department of Pharmaceutical Sciences Utrecht University Utrecht The Netherlands; ^5^ Department of Medical Oncology and Clinical Pharmacology The Netherlands Cancer Institute ‐ Antoni van Leeuwenhoek Amsterdam The Netherlands; ^6^ Department of Clinical Pharmacy University Medical Center Utrecht Utrecht University Utrecht The Netherlands; ^7^ Department of Pharmacology Princess Máxima Center for Pediatric Oncology Utrecht The Netherlands

**Keywords:** body composition, capecitabine, pharmacokinetics, skeletal muscle mass

## Abstract

**Background:**

A low skeletal muscle mass (SMM) has been associated with increased toxicity and shorter survival in cancer patients treated with capecitabine, an oral prodrug of 5‐fluorouracil (5‐FU). Capecitabine and its metabolites are highly water‐soluble and, therefore, more likely to distribute to lean tissues. The pharmacokinetics (PK) in patients with a low SMM could be changed, for example, by reaching higher maximum plasma concentrations. In this study, we aimed to examine whether the association between a low SMM and increased toxicity and shorter survival could be explained by altered PK of capecitabine and its metabolites.

**Methods:**

Previously, a population PK model of capecitabine and metabolites in patients with solid tumors was developed. In our analysis, we included patients from this previous analysis for which evaluable abdominal computed tomography (CT)‐scans were available. SMM was measured on CT‐scans, by single slice evaluation at the third lumbar vertebra, using the Slice‐o‐Matic software. The previously developed population PK model was extended with SMM as a covariate, to assess the association between SMM and capecitabine and metabolite PK.

**Results:**

PK and SMM data were available from 151 cancer patients with solid tumors. From the included patients, 55% had a low SMM. No relevant relationships were found between SMM and the PK parameters of capecitabine and, the active and toxic metabolite, 5‐FU. SMM only correlated with the PK of the, most hydrophilic, but inactive and non‐toxic, metabolite α‐fluoro‐β‐alanine (FBAL). Patients with a low SMM had a smaller apparent volume of distribution and lower apparent clearance of FBAL.

**Conclusions:**

No alterations in PK of capecitabine and the active and toxic metabolite 5‐FU were observed in patients with a low SMM. Therefore, the previously identified increased toxicity and shorter survival in patients with a low SMM, could not be explained by changes in pharmacokinetic characteristics of capecitabine and metabolites.

## Graphical abstract

A low skeletal muscle mass (SMM) has been associated with increased toxicity and shorter survival in cancer patients treated with capecitabine, an oral prodrug of 5‐fluorouracil (5‐FU). No alterations in PK of capecitabine and the active and toxic metabolite 5‐FU were observed in patients with a low SMM in our study. Therefore the previously identified increased toxicity and shorter survival in patients with a low SMM, could not be explained by changes in pharmacokinetic characteristics of capecitabine and metabolites.

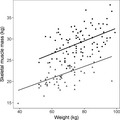

## 1 INTRODUCTION

The anti‐cancer drug capecitabine is used for the treatment of colorectal, breast, and gastric cancer.^1^ Capecitabine is metabolized through conversion to 5'‐deoxy‐5‐fluorocytidine (dFCR), and 5'‐deoxy‐5‐fluorouridine (dFUR), respectively, before it forms the pharmacologically active metabolite 5‐fluorouracil (5‐FU).^2^ 5‐FU is further activated by forming nucleotides intracellularly, and finally converted to the inactive metabolite α‐fluoro‐β‐alanine (FBAL), which is renally excreted.^1^, ^2^, ^3^

A major problem for capecitabine treatment is that more than 40% of the patients experience severe toxicity, when combined with other chemotherapy this number is even higher.^4^, ^5^, ^6^ The most common capecitabine‐induced severe toxicities include diarrhea, vomiting, and hand‐foot syndrome.^4^ Recently, Kurk et al. found that colorectal patients who experience skeletal muscle mass (SMM) loss during treatment with different combinations of palliative systemic treatment regimens, including capecitabine, were at increased risk of developing severe toxicity (relative risk 1.29), and shorter survival was observed (hazard ratio 1.19 or 1.54 dependent on treatment phase).^5^, ^7^ In these studies, Kurk et al. hypothesized that altered drug pharmacokinetics (PK) may contribute to the observed increased toxicity and reduced survival.^5^, ^7^

Several population pharmacokinetic studies have been performed to study the PK of capecitabine and metabolites. Results of these studies demonstrated that there is no clinically relevant correlation between several body size measures such as body surface area (BSA) or body weight and PK of capecitabine and metabolites.^8^, ^9^, ^10^

Since capecitabine and its metabolites are highly water‐soluble, these compounds will distribute mainly to non‐lipid tissues such as muscle tissue.^11^ A low SMM, which is common in cancer patients, may, therefore, result in a smaller volume of distribution and potentially lead to higher maximum plasma levels (which was for example described for the beta‐blocker bisoprolol).^2^, ^12^ Higher maximum plasma levels could be the cause of increased toxicity; a shorter survival could be caused by dose adjustments, dose delay, or early discontinuation of treatment due to toxicity.

We hypothesized that the increased risk of severe capecitabine‐induced toxicity and shorter survival in patients with a low SMM may be the result of an altered distribution of capecitabine and metabolites. Additionally, patients with a low SMM may be less fit, potentially resulting in a reduced cardiac output, which might be associated with reduced clearance of the drug and metabolites.^13^

Measurement of SMM on computed tomography (CT)‐scans, magnetic resonance imaging (MRI), or dual‐energy X‐ray absorptiometry (DXA) analysis is thought to be the most accurate way of estimating SMM.^14^ Since CT‐scans are available for most cancer patients in routine care, CT‐scans are usually used to assess SMM in a population of cancer patients.

The primary aim of the current study was to examine the association between SMM and capecitabine and metabolite PK in a heterogeneous patient population, which might explain the previously found increased toxicity and shorter survival in colorectal cancer patients with a low SMM.

## 2 METHODS

### 2.1 Study population

Our research group previously published a population PK model of capecitabine and metabolites in patients with solid tumors who were treated with capecitabine‐based chemotherapy with or without radiotherapy.^15^ For these patients, first, availability of a CT‐scan in the 5 months before sample collection for PK analysis was checked (if more than one scan was available, the last scan before sample collection was chosen; PK analysis was also performed only with the patients with a difference in time between CT‐scan and PK sampling of less than 2 months). Second, the evaluability of CT‐scans was verified by screening for deviations on the CT‐scans, which would make measurement of SMM impossible (e.g., radiation artifacts). Third, the availability of height and weight was checked. All patients for which evaluable CT‐scans, height, and weight were available were included in the analysis. The study was conducted in accordance with Good Clinical Practice and the Declaration of Helsinki and was approved by the local Medical Ethics Committee. All patients provided written informed consent prior to enrollment in the study.

### 2.2 Measurement skeletal muscle mass

The SMM was measured by using the Slice‐o‐Matic software (version 5.0; TomoVision). First, a slice at the third lumbar level (L3) was selected on the CT‐scan, because the skeletal muscle area at a single slice at the L3 level highly correlates with total body SMM (*r* ^2^ = 0.86).^14^ The first slice where both transverse processes and the spinous process were visible was chosen. The skeletal muscle area on the L3 slice was demarcated using thresholds of −29 to 150 Hounsfield Units (HU). Slice selection and demarcation were performed by a single trained analyst. SMM was calculated from the skeletal muscle area at L3 with the following equations (Equations 1 and 2)^16^, ^17^:

(1) $$Skeletal\;muscle\;volume\;\left(L\right)=0.166L/{\mathrm{cm}}^{2}\times skeletal\;muscle\;area\;in\;{\mathrm{cm}}^{2}+2.142\;L$$

(2) $$SMM\;\left(kg\right)=skeletal\;muscle\;volume\;in\;L\times 1.06g/{\mathrm{cm}}^{3}$$

To determine whether patients had a low SMM, the following threshold values were used. For males an SMM <26.8 kg if BMI <25 kg/m^2^, and an SMM <32.5 kg if BMI ≥25 kg/m^2^; for females an SMM <22.6 kg for any BMI. These threshold values were based on the skeletal muscle index (skeletal muscle area divided by the squared height of the patient) threshold values published by Martin et al.^18^

Furthermore, fat‐free mass (FFM) was calculated to compare with the measured SMM. The equations of Janmahasatian et al. (Equations 3 and 4) were used to calculate FFM.^19^, ^20^, ^21^, ^22^

(3) $$FFM\;\left(in\;males\right)=\frac{9.27\;\times \;10^{3}\;\times \;weight}{6.68\;\times \;10^{3}\;+\;216\;\times \;BMI}$$

(4) $$FFM\;\left(in\;females\right)=\frac{9.27\;\times \;10^{3}\;\times \;weight}{8.78\;\times \;10^{3}\;+\;244\;\times \;BMI}$$

In these equations, weight is the total body weight in kg of a patient and BMI is the body mass index (weight/height^2^; weight in kg and height in m).

The correlations between SMM, and weight and FFM were tested using the Pearson correlation coefficient.

### 2.3 Population pharmacokinetic modeling

A population PK model of capecitabine and its four metabolites was previously developed by our research group.^15^ The developed model was based on data from seven clinical studies, in which pharmacokinetic samples were taken on day 1 or day 22 and 43 of treatment with capecitabine. Rich sampling schedules were used with samples taken between 0 and 8 h after intake of capecitabine. The structural, covariate (which included DPYD*2A and gastric surgery status), and random effects model from the published model were maintained, but parameter estimates were re‐estimated. Various body composition descriptors were evaluated as covariates in this existing model: weight, SMM, and FFM. Age and gender were initially removed from the covariate model, because theoretically the effects of age and gender can be caused by a lower SMM in older people and women. After evaluation of the candidate covariates weight, SMM, and FFM, the effects of age and gender on metabolite PK were assessed again.

Weight, SMM, and FFM were separately evaluated as covariates on apparent clearance (CL/F), apparent volume of distribution of the central compartment (V_c_/F), intercompartmental clearance (Q), and volume of distribution of the peripheral compartment (V_p_) or the elimination rate constant (k) of capecitabine, dFCR, dFUR, 5‐FU, and FBAL.

The effects of body composition descriptors weight, SMM, and FFM on CL/F, V_c_/F, Q, and V_p_ were estimated pairwise as follows (Equations 5 and 6)^23^, ^24^, ^25^, ^26^:

(5) $$CL={\mathrm{CL}}_{\mathrm{baseline}}\times {\left(\frac{body\;composition}{median\;body\;composition}\right)}^{0.75}$$

(6) $$V=V_{\mathrm{baseline}}\times {\left(\frac{body\;composition}{median\;body\;composition}\right)}^{1}$$

In which CL and V are the body composition adjusted clearance and volume of distribution, respectively. CL_baseline_ and V_baseline_ are the baseline estimates for clearance and volume of distribution, respectively.

In case of dFUR and 5‐FU, only k's were previously identifiable. For these metabolites, the effects of body composition descriptors weight, SMM, and FFM were estimated as follows (Equation 7):

(7) $$k=k_{\mathrm{baseline}}\times {\left(\frac{body\;composition}{median\;body\;composition}\right)}^{‐0.25}$$

In which k is the body composition adjusted elimination rate constant, and k_baseline_ is the baseline estimate for the elimination rate constant.

Based on the theory of allometric scaling, fixed coefficients were used, when testing weight, SMM, and FFM as covariates.^23^ In addition, the coefficients were also estimated, to further investigate the relationship between SMM and PK of capecitabine and metabolites.

### 2.4 Model evaluation

For model evaluation, the likelihood ratio test was used and the model fit was assessed (indicated by successful minimization, parameter precision [obtained using the $COVARIANCE option of NONMEM®], and a drop in inter‐individual variability). Formal statistical testing of the effect of SMM on PK could not be performed, since the models using fixed allometric relationships were nonhierarchical compared to the models lacking these associations. Therefore, an arbitrary drop of 15 points in minus twice the log‐likelihood value was considered relevant. For models in which the exponents of the allometric relationships were estimated, formal statistical testing could be performed using the likelihood ratio test with the degrees of freedom equal to the number of included relationships. To account for multiple testing, a *p*‐value of 0.005 was used.^27^

## 3 RESULTS

**FIGURE 1:**
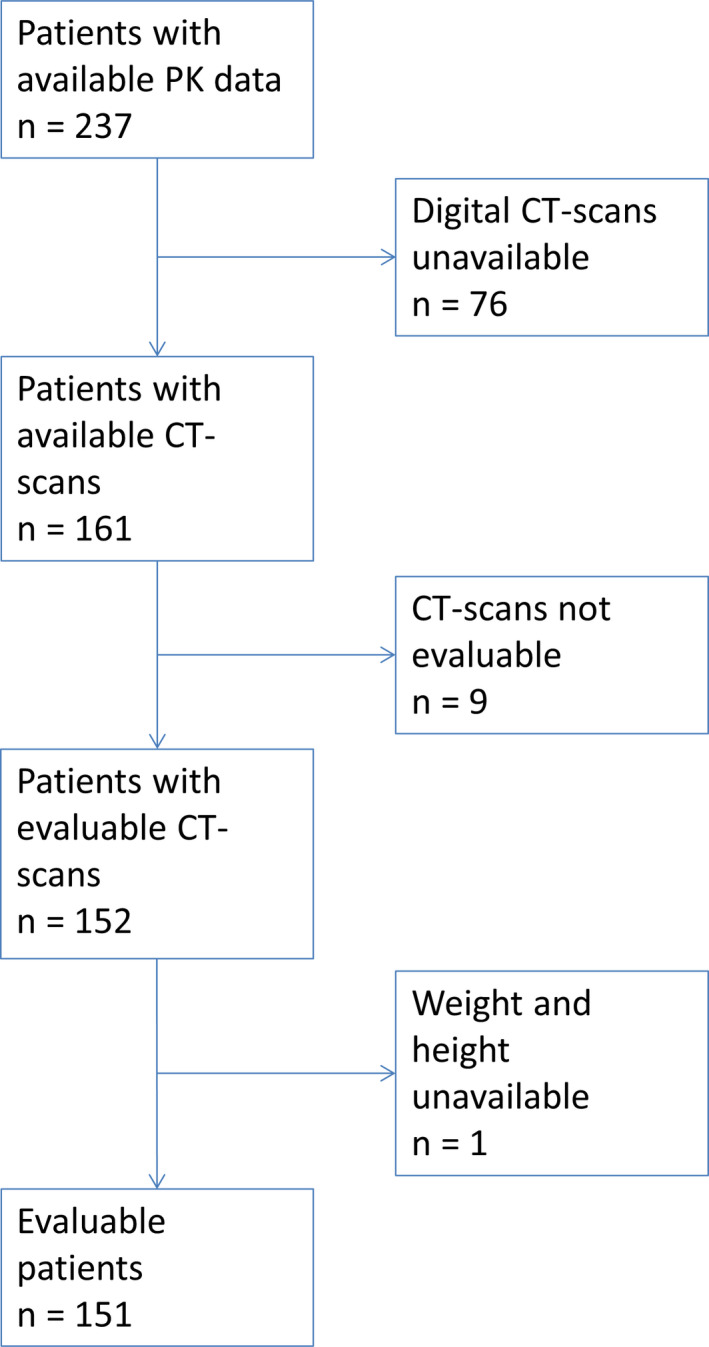
Schematic overview of patient selection, starting with the patients included in the population PK model published by Jacobs et al.^15^ CT‐scan, computed tomography‐scan; PK, pharmacokinetic

A schematic overview of the patient selection is shown in Figure 1. For nine patients, the CT‐scans were not evaluable, due to anatomical abnormalities, radiation artifacts or unavailability of scan data of the L3 slice. One patient was not evaluable because height and weight data were missing. Ultimately, full data were available for 151 patients. In Table 1, the patient characteristics are shown. From the included patients, 55% had a low SMM. The time between CT‐scan and pharmacokinetic sampling had a median of less than a month, and was shorter than 2 months in 93% of patients, with exceptions to a maximum of 154 days.

**TABLE 1:** Patient characteristics (*n* = 151)

Characteristic	*n* (%) or median (range)
Capecitabine dose (mg)	1650 (300–2600)
Gender	
Male	93 (62%)
Female	58 (38%)
Age (years)	58 (31–77)
DPYD*2A	
Wildtype	139 (92%)
Heterozygous mutant	12 (8%)
Gastric surgery	
No gastrectomy	103 (68%)
Total gastrectomy	15 (10%)
Partial gastrectomy	24 (16%)
Esophagogastrectomy	9 (6%)
Height (cm)	174 (152–201)
Weight (kg)	73 (39–99)
BSA (m^2^)	1.9 (1.3–2.3)
BMI (kg/m^2^)	24 (16–35)
Skeletal muscle mass (kg)^a^	27 (15–38)
Fat‐free mass (kg)^b^	55 (29–73)
Low skeletal muscle mass^c^	
Yes	84 (56%)
No	67 (44%)
Time between CT‐scan and PK sampling (days)	26 (0–154)

Abbreviation: CT‐scan, computed tomography‐scan; *DPYD*, dihydropyrimidine dehydrogenase; PK, pharmacokinetic.

^a^ Skeletal muscle mass as measured on CT‐scans.

^b^ Fat‐free mass as calculated by the formulas of Janmahasatian et al.^20^

^c^ A low skeletal muscle mass was for males defined as an SMM <26.8 kg if BMI <25 kg/m^2^ and as SMM <32.5 kg if BMI ≥25 kg/m^2^ (calculated with a median height of 180 cm), and for females as an SMM <22.6 kg for any BMI (calculated with a median height of 168 cm).^18^

**FIGURE 2:**
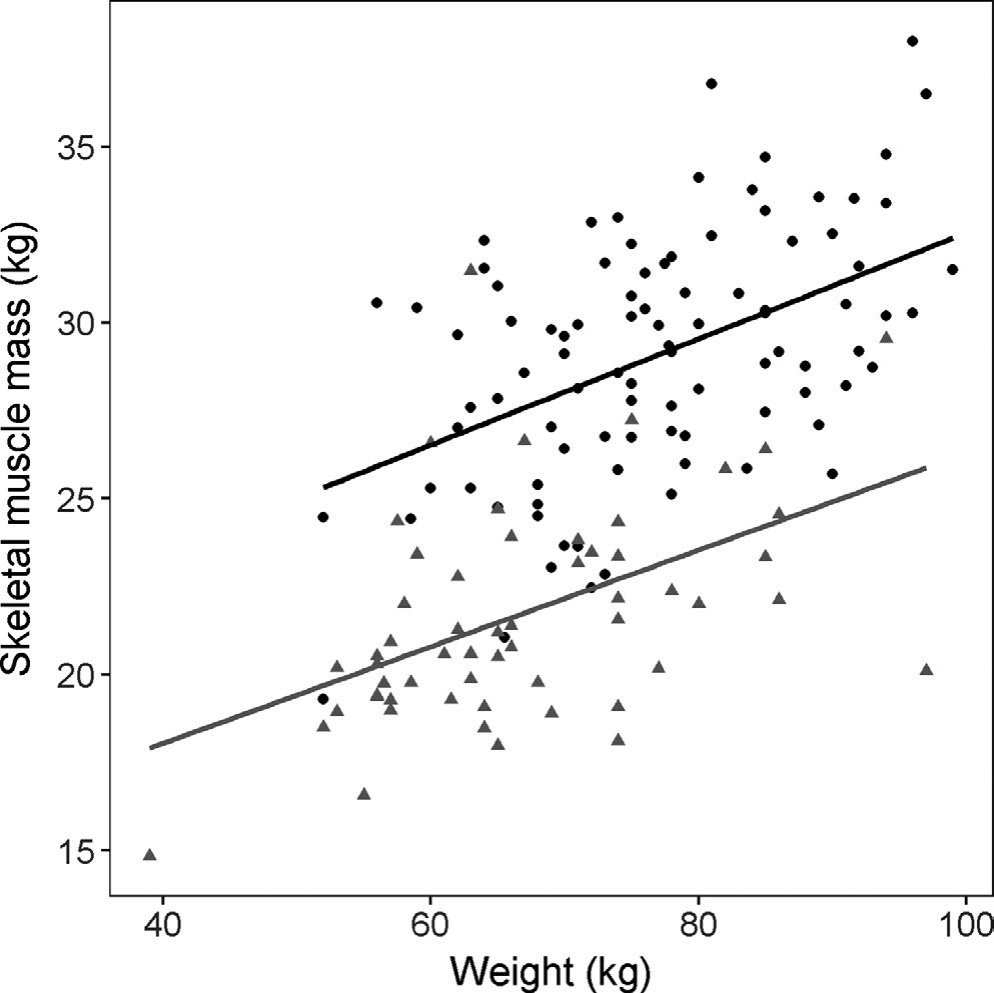
Correlation between weight and skeletal muscle mass, separated by gender. Males are displayed by black circles and females by grey triangles. Correlation coefficient males: *R* = 0.49, *p* < 0.005; females: *R* = 0.47, *p* < 0.005

**FIGURE 3:**
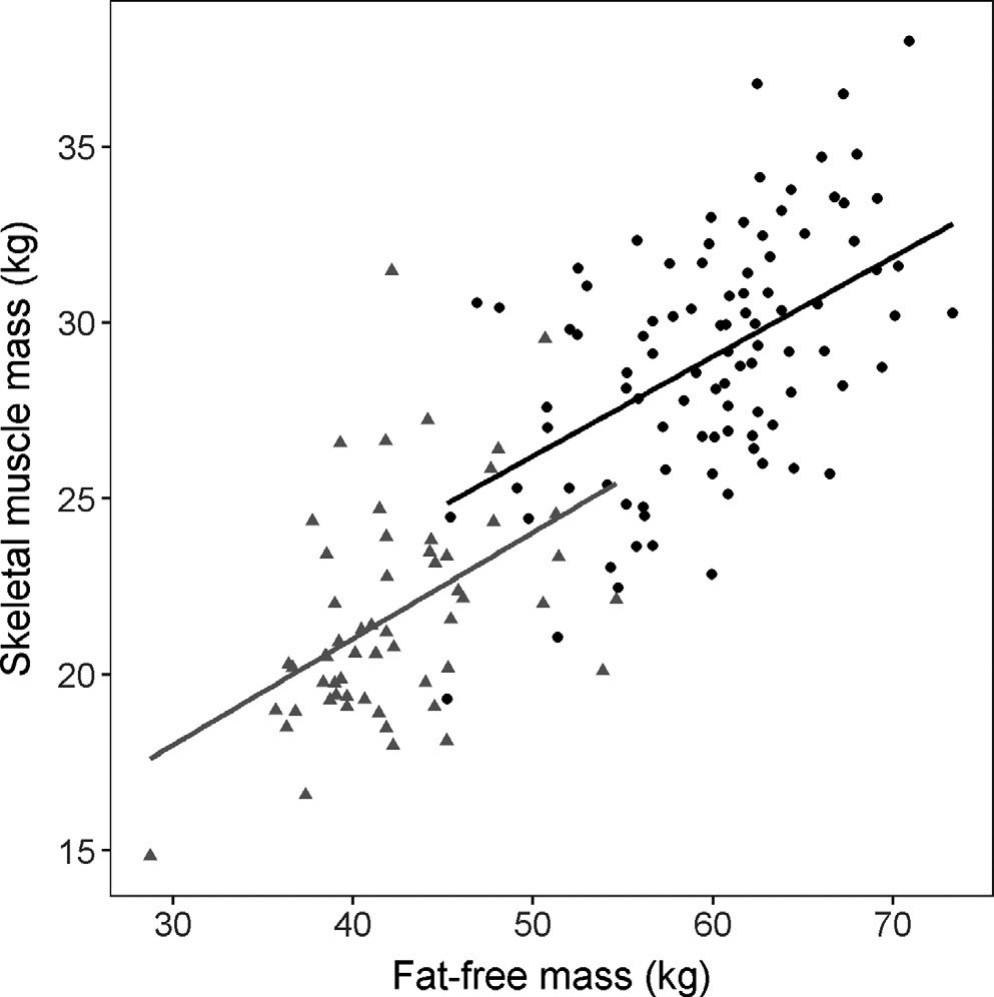
Correlation between calculated fat‐free mass and skeletal muscle mass, separated by gender. Males are displayed by black circles and females by grey triangles. Correlation coefficient males: *R* = 0.53, *p* < 0.005; females: *R* = 0.45, *p* < 0.005

SMM, weight, and FFM were significantly but poorly correlated (Pearson correlation coefficients *r* = 0.5, Figures 2 and 3). Therefore, weight, SMM, and FFM were tested in separate PK models.

First, the PK model was extended with one of the three parameters for body composition (weight, SMM or FFM) as a covariate on the PK parameters (CL/F, V_c_/F, Q, V_p_, k) of capecitabine and its four metabolites all at once, with fixed coefficients. In all cases, a small increase in minus twice log‐likelihood value was found, demonstrating the lack of an overall effect of body size measures on the PK.

Second, weight, SMM, and FFM were separately added as potential covariates for the PK of capecitabine, dFCR, dFUR, and 5‐FU. Also here, no relevant relationships were found between PK parameters and the different measures for body size (no relationships reaching the 15 points minus twice log‐likelihood value decrease threshold).

Finally, weight, SMM, and FFM were introduced as potential covariates on CL/F, V_c_/F, Q, and V_p_ of FBAL. Addition of weight as a potential covariate increased the minus twice log‐likelihood value by 5 points. Addition of SMM and FFM resulted in a drop in minus twice the log‐likelihood value of 28 points for SMM and 24 points for FFM, suggesting a relevant relationship with the pharmacokinetic parameters.

**FIGURE 4:**
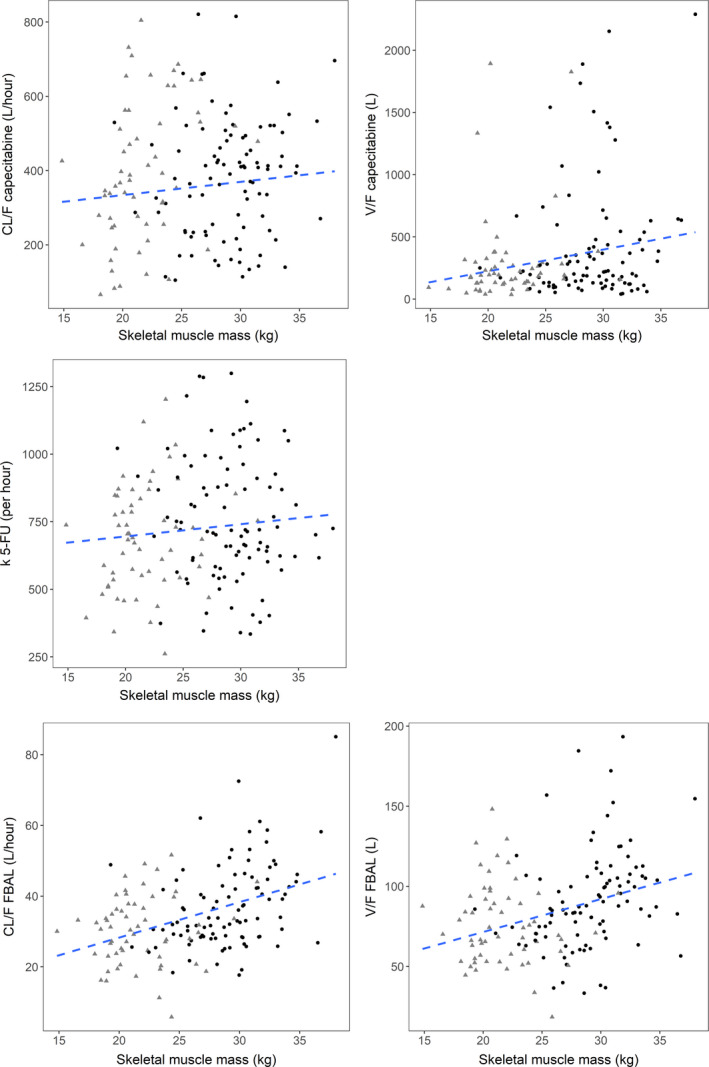
Relationships between skeletal muscle mass and the individual pharmacokinetic parameters of capecitabine, 5‐FU and FBAL. Males are displayed by black circles and females by grey triangles including a linear regression line (dashed line). 5‐FU, 5‐fluorouracil; CL/F, apparent clearance; FBAL, α‐fluoro‐β‐alanine; k, elimination rate constant; V/F, apparent volume of distribution central compartment

In Table 2, the results for the inclusion of weight, SMM, and FFM, with fixed coefficients, in the PK model are summarized. Figure 4 shows the relationships between SMM and the individual PK parameters of capecitabine, 5‐FU and FBAL, before SMM was added as a covariate on the corresponding compound. In case a relationship between SMM and the pharmacokinetic parameters exists, the figure should show a correlation.

**TABLE 2:** Effect of body composition, age, and gender on the pharmacokinetic model using the likelihood ratio test

Model	Δ minus twice log‐likelihood value relative to the baseline model
Covariate effect on CL/F, V_c_/F, Q, and V_p_ of capecitabine, dFCR, and FBAL, and on k of dFUR and 5‐FU	
Weight	+7
SMM	+12
FFM	+4
Covariate effect on CL/F, V_c_/F, Q, and V_p_ of capecitabine	
Weight	−8
SMM	−1
FFM	−1
Covariate effect on CL/F, V_c_/F, Q, and V_p_ of dFCR	
Weight	−8
SMM	+13
FFM	+3
Covariate effect on k of dFUR	
Weight	+8
SMM	+6
FFM	+8
Covariate effect on k of 5‐FU	
Weight	+6
SMM	+5
FFM	+8
Covariate effect on CL/F, V_c_/F, Q, and V_p_ of FBAL	
Weight	+5
SMM	−28
FFM	−24
Covariate effect on CL/F of FBAL	
Age and gender	−51
Covariate effect of SMM on CL/F, V_c_/F, Q, and V_p_ of FBAL, combined with covariate effect of	
Age + gender on CL/F of FBAL	−50
Age on CL/F of FBAL	−50
Gender on CL/F of FBAL	−27

Note

Coefficients were fixed based on the theory of allometric scaling. To calculate the difference in the minus twice log‐likelihood value, the baseline model was used as a comparator. No *p*‐values are shown because the tested models were nonhierarchical and therefore no formal statistical testing could be performed. A positive difference in minus twice log‐likelihood value indicates a worse model fit, and a negative difference indicates a better model fit.

Abbreviations: 5‐FU, 5‐fluorouracil; CL/F, apparent clearance; dFCR, 5'‐deoxy‐5‐fluorocytidine; dFUR, 5'‐deoxy‐5‐fluorouridine; FBAL, α‐fluoro‐β‐alanine; FFM, fat‐free mass; k, elimination rate constant; Q, intercompartmental clearance; SMM, skeletal muscle mass; V_c_/F, apparent volume of distribution for the central compartment; V_p_, volume of distribution of the peripheral compartment.

Next, the exponents of the allometric relationships were estimated for SMM. In comparison to the results with a fixed coefficient, the minus twice log‐likelihood value was between 5 and 21 points lower if the coefficient was estimated. The drop in minus twice log‐likelihood value of 21 points was observed for dFCR, but compared to the baseline model without SMM as a covariate, it was only a drop of 7 points and therefore not considered clinically relevant. The estimated coefficient for capecitabine was 0.271 for CL/F and Q, and 1.5 for V_c_/F and V_p_. A coefficient of 0.0815 was estimated for the k of 5‐FU. And the estimated coefficient for FBAL was 0.782 for CL/F and Q, and 0.559 for V_c_/F and V_p_. Except for the coefficient on V of capecitabine, the estimated coefficients were small (in allometric scaling 0.75 is used for CL and 1 for V). To determine if the difference in the coefficient on V of capecitabine was significant, SMM was added as a covariate on V of capecitabine only, instead of adding it on V and CL of capecitabine simultaneously. This resulted in a significant different coefficient of 1.25 (*p* < 0.005, drop in minus twice log‐likelihood value of 9 points), but with a large relative standard error (RSE) and 95% confidence interval of 42% and 0.43–2.07, respectively. To conclude, the estimation of the coefficients did not result in a different interpretation of the outcomes.

In the previously published model age and gender were added as covariates on the CL of FBAL, which were initially removed in our analysis.^15^ After the introduction of SMM as a covariate on CL/F, V_c_/F, Q, and V_p_ of FBAL, the addition of gender did not result in a significant improvement of the model anymore. This final model (with SMM as a covariate on CL/F, V_c_/F, Q, and V_p_ of FBAL, and age as a covariate on CL/F of FBAL) was also tested without the patients with a difference in time between CT‐scan and PK sampling of more than 2 months, which did not lead to a difference in the predicted parameter estimates.

## 4 DISCUSSION

In patients treated with capecitabine, often given in combination with other toxic chemotherapy or targeted therapy, a low SMM has been associated with more treatment‐related toxicity and shorter survival.^5^, ^28^, ^29^, ^30^ The primary aim of our study was to investigate whether this association could be explained by altered pharmacokinetics, such as higher maximum plasma concentrations of capecitabine and/or its metabolites due to a low SMM.

The results of our study showed no effects of SMM on the PK of capecitabine and its active metabolite 5‐FU. However, SMM was associated with PK of the most hydrophilic metabolite FBAL. Previously, Gieschke et al. found that there is no relationship between the area under the concentration‐time curve (AUC; indicator of exposure to the drug) of FBAL and treatment‐related grade 3–4 adverse events, including treatment‐related grade 3–4 diarrhea, grade 3 hand–foot syndrome, and grade 3–4 hyperbilirubinemia.^9^ Only a correlation between the AUC of FBAL and diarrhea was found, but this was probably due to FBAL being a marker for the amount of 5‐FU formed.^9^ Also, there was no relation between the AUC of FBAL and time to progression and survival.^9^ Therefore, the identified effects of SMM on PK of FBAL are considered clinically irrelevant. Our results, thus, do not support the hypothesis that patients with a low SMM would show relatively low values for CL and/or V of capecitabine and metabolites, which thereby would provide an explanation for the increased toxicity and decreased survival in patients with a low SMM.^9^, ^31^

Previously, Gusella et al. studied the relationship between body composition and PK of intravenously administered 5‐FU, in 43 patients with colorectal cancer.^32^ Significant but poor correlations between total body water and CL (*r* ^2^ = 0.15), total body water and V (*r* ^2^ = 0.16), FFM and CL (*r* ^2^ = 0.17), and FFM and V (*r* ^2^ = 0.17) were found. But also poor correlations were found between BSA and CL (*r* ^2^ = 0.12), body weight and CL (*r* ^2^ = 0.21), and between body weight and V (*r* ^2^ = 0.18). We could not reproduce these findings in this much larger study for capecitabine, in which we included 151 patients. A major difference between our study and the study of Gusella et al. was the administration of the pro‐drug capecitabine instead of intravenous administration of 5‐FU. It might be expected that variability in PK is much larger after oral administration of capecitabine than after intravenous administration of 5‐FU, because of variability in absorption time and bioavailability of capecitabine. Indeed, inter‐individual variability in PK parameters of capecitabine and metabolites was high. Therefore, it might be possible that a relatively small effect of SMM on the PK of capecitabine, 5‐FU, and metabolites remains undetected in the overall large variability in PK. If so, the absence of significant relationships in our large study strongly suggests that these potential effects are only of minor clinical relevance.

An advantage over the study of Gusella et al. is that in our study body composition was measured on CT‐scans. There are different ways of determining body composition, based on different physical and biological principles. In the study of Gusella et al., bioelectrical impedance analysis (BIA) was used to determine fat‐free mass, in which the rate of a low‐voltage electrical current traveling through the body is measured.^32^ This technique is simple and safe, but not very accurate because it is dependent on the hydration of the body (total body water is measured and FFM is calculated based on the assumption that 73% of the FFM consists of water).^33^ Imaging methods are considered the most accurate to measure body composition and can therefore be seen as “gold standards”.^14^, ^19^, ^33^ Since CT‐scans are available for most cancer patients in routine care, patients are not exposed to additional radiation for measurement of body composition using these scans. The most accurate results are obtained by using CT‐scans just before the start of treatment with capecitabine.^34^ Due to the retrospective nature of our study, the range in time between CT‐scans and PK sampling was up to 5 months, but with a median of less than 1 month, and it was shorter than 2 months in 93% of patients. The final model (with SMM as a covariate on CL and V of FBAL, and age as a covariate on CL of FBAL) was also tested without the ten patients with a range in time between CT‐scans and PK sampling of more than 2 months, which did not lead to a difference in parameter estimates predicted by the model.

It could be seen as a limitation of our study that allometric scaling was applied when testing SMM as a covariate. Based on the theory of allometric scaling, which is a generally accepted theory for scaling of PK parameters between different body sizes, a fixed coefficient of 0.75 was used, when weight, SMM, and FFM were tested as potential covariates on CL and Q (Equation 5). The basis of allometric scaling is a slope of 0.75 when body weight is plotted against basal metabolic rate, which most likely also applies to SMM.^23^, ^24^, ^25^, ^26^ To further investigate the relationship between SMM and PK of capecitabine and metabolites, the exponents in the relationships were also estimated. Overall, these exponents were small and with large confidence intervals, indicating that indeed no relationship between SMM and PK could be found.

## 5 CONCLUSIONS

To conclude, results of the analyses demonstrated that PK of capecitabine and its metabolite 5‐FU are not associated with SMM. Therefore, alterations in capecitabine and metabolite PK do not provide an explanation for increased toxicity and decreased survival in patients with a low SMM.

## CONFLICT OF INTEREST

J.H. Beijnen (partly) holds a patent on oral taxane formulations and is a (part time) employee and stockholder of Modra Pharmaceuticals, a spin‐out company developing oral taxanes. Not related to the manuscript. N. Steeghs provided consultation or attended advisory boards for AIMM Therapeutics, Boehringer Ingelheim, Ellipses Pharma. N. Steeghs received research grants for the institute from AB Science, Abbvie, Actuate Therapeutics, Amgen, Array, AstraZeneca/MedImmune, Bayer, Blueprint Medicines, Boehringer Ingelheim, Bristol‐Myers Squibb, Cantargia, Cytovation, Deciphera, Genentech/Roche, GlaxoSmithKline, Incyte, InteRNA, Lilly, Merck Sharp & Dohme, Merus, Novartis, Pfizer, Pierre Fabre, Roche, Sanofi, Taiho, Takeda (outside the submitted work). The other authors indicated no financial disclosures.

## ETHICS STATEMENT

The study was conducted in accordance with Good Clinical Practice and the Declaration of Helsinki and was approved by the local Medical Ethics Committee. All patients provided written informed consent prior to enrollment in the study.

## Data Availability

The data that support the findings of this study are available from the corresponding author upon reasonable request.
